# Effects of carbon nanotubes on expanded glass and silica aerogel based lightweight concrete

**DOI:** 10.1038/s41598-021-81665-y

**Published:** 2021-01-22

**Authors:** Suman Kumar Adhikary, Žymantas Rudžionis, Simona Tučkutė, Deepankar Kumar Ashish

**Affiliations:** 1 Faculty of Civil Engineering and Architecture, Kaunas University of Technology, 44249 Kaunas, Lithuania; 2 Center for Hydrogen Energy Technologies, Lithuanian Energy Institute, Breslaujos st. 3, 44403 Kaunas, Lithuania; 3 Civil Engineering Department, Maharaja Agrasen Institute of Technology, Maharaja Agrasen University, Baddi, 174 103 India; 4 Civil Engineering Department, Punjab Engineering College (Deemed to be University), Chandigarh, 160012 India

**Keywords:** Civil engineering, Carbon nanotubes and fullerenes

## Abstract

This study is aimed to investigate the effect of carbon nanotubes on the properties of lightweight aggregate concrete containing expanded glass and silica aerogel. Combinations of expanded glass (55%) and hydrophobic silica aerogel particles (45%) were used as lightweight aggregates. Carbon nanotubes were sonicated in the water with polycarboxylate superplasticizer by ultrasonication energy for 3 min. Study results show that incorporating multi-wall carbon nanotubes significantly influences the compressive strength and microstructural performance of aerogel based lightweight concrete. The addition of carbon nanotubes gained almost 41% improvement in compressive strength. SEM image of lightweight concrete shows a homogeneous dispersal of carbon nanotubes within the concrete structure. SEM image of the composite shows presence of C–S–H gel surrounding the carbon nanotubes, which confirms the cites of nanotubes for the higher growth of C–S–H gel. Besides, agglomeration of carbon nanotubes and the presence of ettringites was observed in the transition zone between the silica aerogel and cementitious materials. Additionally, flowability, water absorption, microscopy, X-ray powder diffraction, and semi-adiabatic calorimetry results were analyzed in this study.

## Introduction

Nowadays, the use of carbon nanotubes in cementitious materials is getting attention for the improvement of physical and mechanical characteristics. Carbon nanotubes (CNTs) are an allotrope of carbon composed of coaxial hexagonal carbon rings, cylindrical in shape having around 132,000,000:1 length–diameter ratio. The nanoscale diameters and smooth surfaces of CNT’s could affect the early age hydration of cementitious materials^1^. Carbon nanotubes can be categorized into single-wall carbon nanotube (SWCNT) and multiwall carbon nanotube (MWCNT). Inclusion of small doses of MWCNT can effectively improve the mechanical properties of cementitious composites improving early age and long-term durability^2^. The reinforcing efficiency of carbon nanotubes can be influenced by several parameters such as type of CNTs^3^, the concentration of CNT^4^, dispersion surfactants^5^, treatment of CNTs^6,7^, dispersion technique^8^, the interaction with cementitious materials and bond strength^9^, the water–cement ratio^10,11^ and geometry of CNTs^12,13^. Zou et al.^8^ and Collins et al.^10^ reported that ultrasonication energy and polycarboxylate-based superplasticizer could significantly influence the mechanical and microstructural properties of CNT incorporated cementitious composites by optimally dispersing the CNTs within a concrete structure^8,10^. Han et al.^14^ reported that polycarboxylate-based superplasticizer plays a double dispersion mechanism to disperse the cement and CNTs within the composite. Without proper dispersion technique, agglomeration of CNTs can be noticed in the concrete structure due to the strong van der Waals forces and can influence the mechanical and microstructural properties. The improvement in cementitious composites were found different for cement mortar, cement paste, and concrete. The highest improvement in compressive and flexural strength was observed 83.33%^15^ and 30%^16^ for cement paste; ~ 35%^3^ and 28.04%^17^ for mortar; 38.62% and 38.63% for concrete^18^, respectively.

The strength development of cementitious composite also depends on the properties of aggregates and what kind of pozzolanic materials were used. With the increase in the consumption of natural aggregates, researchers are mainly focused on conserving the natural eco-system that leads to the use of lightweight aggregates such as expanded glass aggregates, silica aerogel^19^. Silica aerogel is a lightweight thermal insulating material having low density and high specific surface area, allowing its application in many areas^20^. Expanded glass aggregates is also a thermal conducting material with porous structure^21^. Lightweight aggregates are 25–35% lighter than the conventionally used aggregates^19^. However, the strength properties of lightweight aggregates are also much lower than the conventional aggregates; due to this fact, cement composites utilizing lightweight aggregates generally achieve lower mechanical properties and densities^22^. Mechanical properties of lightweight aggregate concrete also depend on the desired density of the composite; decreasing the density reduces the mechanical properties of composite^23^. Kurpińska and Ferenc^24,25^ investigated the importance of grading of lightweight aggregates. They reported that an optimal graduating of lightweight aggregates could reduce the porosity of lightweight aggregate concrete and improve mechanical performance. Major studies have been presented on the use of lightweight aggregate such as fly ash^26,27^, expanded glass aggregate^28^, silica aerogel^21^ in cement mortar. A study conducted by Yousefi et al.^21^ reported a decrease in compressive strength of cement mortar containing expanded glass aggregates, however inclusion of nano titanium dioxide as nanomaterials increased compressive strength by 39.02%.

Similarly, hydrophobic silica aerogel is also a very lightweight and brittle material, and its incorporation in concrete results in a decrease in mechanical performance^29,30^. In addition, hydrophobic silica aerogel has lower adhesive properties with water-rich cementitious materials. Several researchers observed the separation gaps between the aerogel and cementitious materials in the transition zone^20,31^. CNT is a well-known material for improving mechanical performance and cracks bridging mechanism^32^. The incorporation of CNT not only bridges the cracks and voids; it can also change the microstructure of hydration products^33^. Singh et al.^34^ identified new compounds due to the chemical bonds between the hydrates and carbon nanotubes. Liew et al.^35^, in their study, suggested that CNTs also provide sites for the growth of C–S–H, and CNT can coat the calcium silicate hydrate and provide a larger contact area between the hydration product and CNT. As a result, stronger bonds are created between them, which significantly helps to improve the mechanical properties of cementitious composites^35^.

In this study, several lightweight concrete samples were prepared with different doses of CNTs. Expanded glass aggregates and hydrophobic silica aerogel were used as lightweight aggregates. Microscopy of the composite was deeply analyzed, especially surrounding the hydrophobic aerogel particles. The main aim was to improve the strength property of aerogel concrete by reducing the separation gaps between silica aerogel and cementitious materials. This is the first study that presents the use of CNTs to enhance the compressive strength property of lightweight concrete prepared with expanded glass aggregates and hydrophobic silica aerogel. The study also investigates fluidity, water absorption, and microstructural analysis.

## Results and discussion

### Flowability test of CNT-LWAC

**Figure 1:**
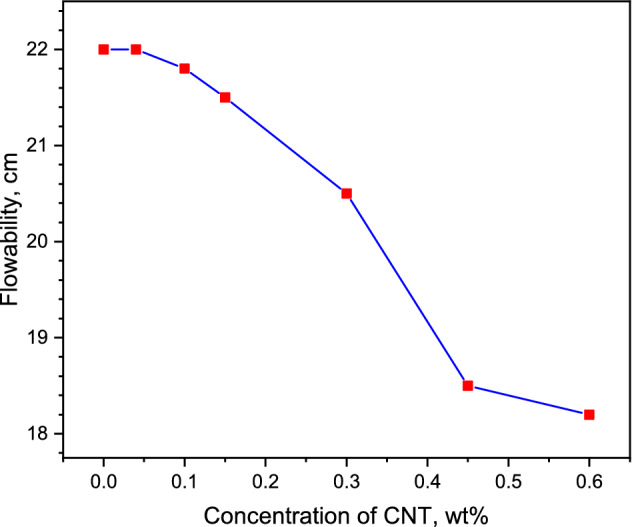
Flowability of sonicated CNT-LWAC specimens.

The flowability of cementitious composites containing CNT depends on several parameters like ultrasonication energy^8^, treatment of CNTs^36^, water-cement ratio^37^, the concentration of CNTs^38^, and types of fine fillers^39^. Mostly studied literature^10,38,40,41^ illustrates that the incorporation of CNTs reduced the flowability of cementitious composites. While there are several studies that indicate the increase in flowability of CNT incorporated cementitious composites^36,39,42^. Besides, the incorporation of silica aerogel also reduces the flowability of cementitious composites^20^. The results of the present study revealed that the flowability of CNT-LWAC was influenced by the quantity of CNT. The flowability of CNT-LWAC decreases with the escalating doses of CNTs. Almost a 17% reduction in flowability was measured for lightweight concrete specimen prepared with 0.6 wt% CNT. The flowability of CNT-LWAC specimens is shown in Fig. 1. The possible reason for the reduction in flowability can be the high surface area and the elongated shape of nanoparticles. Higher doses of superplasticizer can adjust the flowability of the concrete, high specific gravity of CNTs also demands for the large amount of superplasticizer to overcome the intermolecular forces. Besides, nanoparticles enhance the packing density of concrete by filling up the micro and mesopores, significantly influencing the demand for superplasticizer^32^.

### Semi-adiabatic calorimetry test of CNT-LWAC

**Figure 2:**
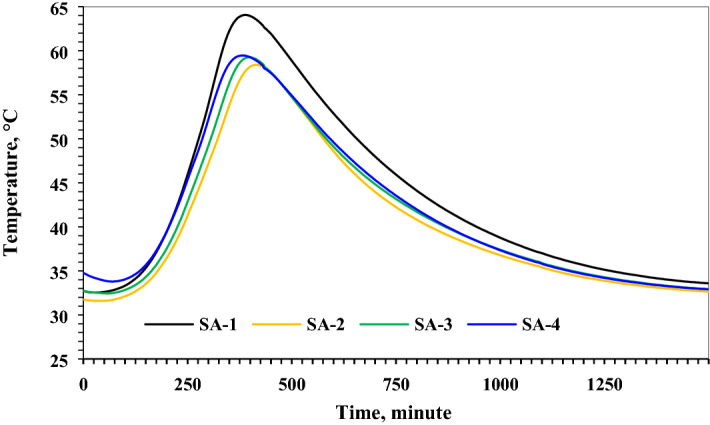
Hydration of cement mortar containing aerogel and sonicated and un-sonicated CNTs.

Figure 2 demonstrates the semi-adiabatic temperature rise of CNT incorporated cement mortar. Study results revealed that the incorporation of silica aerogel slightly retarded the setting time of the cement mortar and produced lower heat of hydration than the plain cement mortar. Study results also confirm the reactivity of silica aerogel with cementitious materials. Similar phenomena were noticed in the previous study^20^. The addition of CNTs to the cement paste having aerogel produced slightly higher heat as indicated in samples SA-3 and SA-4 and shortened setting time compared to sample SA-2. Besides, by increasing the concentration of CNTs, slightly high heat of the exothermic reaction was noticed.

### Strength of CNT-LWAC

**Figure 3:**
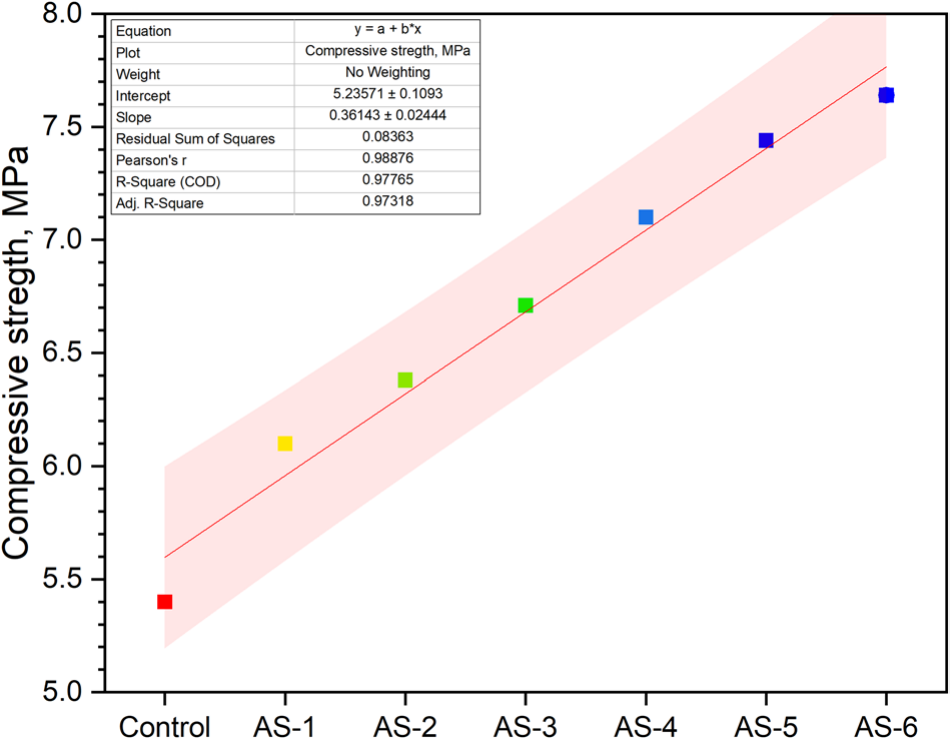
Compressive strength of sonicated CNT-LWAC specimens.

Figure 3 shows the compressive strength of CNT-LWAC specimens. The average compressive strength of the control sample was measured 5.4 MPa. Study results show that the addition of CNTs to the lightweight concrete significantly influences the mechanical strength of concrete. An increase in the concentration of CNTs showed a gradual increase in the compressive strength; however, the sonicated LWAC specimens showed better improvement in compressive strength against the control concrete sample. The compressive strength of the sonicated concrete specimens was increasing by the increasing doses of CNTs. The highest improvement in compressive strength was measured at 41.48% for AS-6 containing 0.60 wt% CNT. As aerogel is a lightweight, fragile material, the literature confirms the reduction in the mechanical performance of aerogel incorporated cementitious composites^43–45^. However, the incorporation of small doses of CNTs to the aerogel based lightweight concrete significantly increased the compressive strength. The increase in compressive strength can be attributed to the nucleating effects and improvement in the microstructure of CNT-LWAC. The C–S–H gel was identified surrounding the CNTs, moreover, hydration products and CNTs fill the micro crack/gaps of the lightweight concrete to provide additional support and increase compressive strength. Detailed discussion is given in section “*Scanning electronic microscopy (SEM) of CNT-LWAC*”. The literature studies confirm that the incorporation of nanoparticles improves the microstructure and provides a denser concrete structure^32^. The confidence intervals for compressive strength are presented in Fig. 3 which shows the example of the straight-line correlation until 0.6% of CNTs use from cement mass. X is the type of CNT-LWAC, and mean compressive strength is given as Y variable values obtained on cubes.

### Water absorption

**Figure 4:**
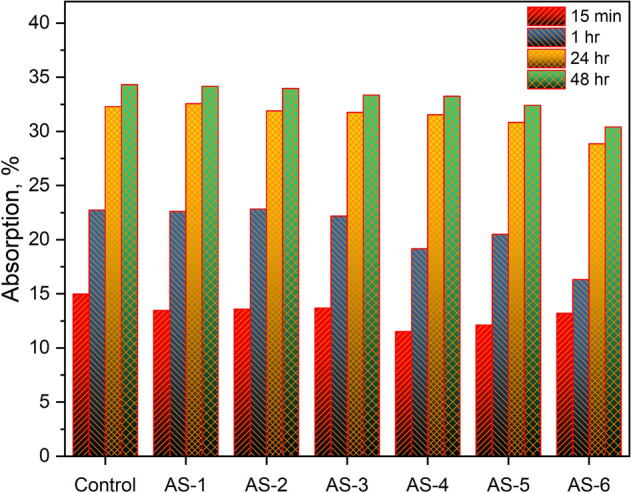
Water absorption of CNT-LWAC specimen.

Figure 4 illustrates the water absorption kinetics of the CNT-LWAC specimens containing up to 0.60 wt% CNTs. Water absorption rate of control sample was measured 14.98%, 22.74%, 32.33% and 34.33% at 15 min, 1 h, 24 h and 48 h that decreased to 13.20%, 16.31%, 28.86% and 30.42% respectively. Madhavi et al.^46^, and Leonavičius et al.^47^ reported that at a lower concentration of CNTs, total pore volume was observed reducing attributed to filling of voids that lead to a reduction in water absorption. According to Leonavičius et al.^47^ and Kordkheili et al.^48^ high concentration of CNTs in concrete results in a more porous structure and reduces the mechanical performance of concrete^47,48^. In the present study, all nanocomposite concrete specimens showed a marginal decrease in water absorption rate by an increase in the concentration of CNTs. The reduction in water absorption can be attributed to the association of CNTs with the lightweight concrete that helped in decreasing the micropores and produced the denser concrete structure. Besides, a comparatively higher water absorption rate was observed than Leonavičius et al.^47^ and Kordkheili et al.^48^ due to the use of expanded glass aggregates in the study. Expanded glass aggregates are porous in structure that can absorb water up to 20–25%, attributed to an increase in water absorption.

### Scanning electronic microscopy (SEM) of CNT-LWAC

**Figure 5:**
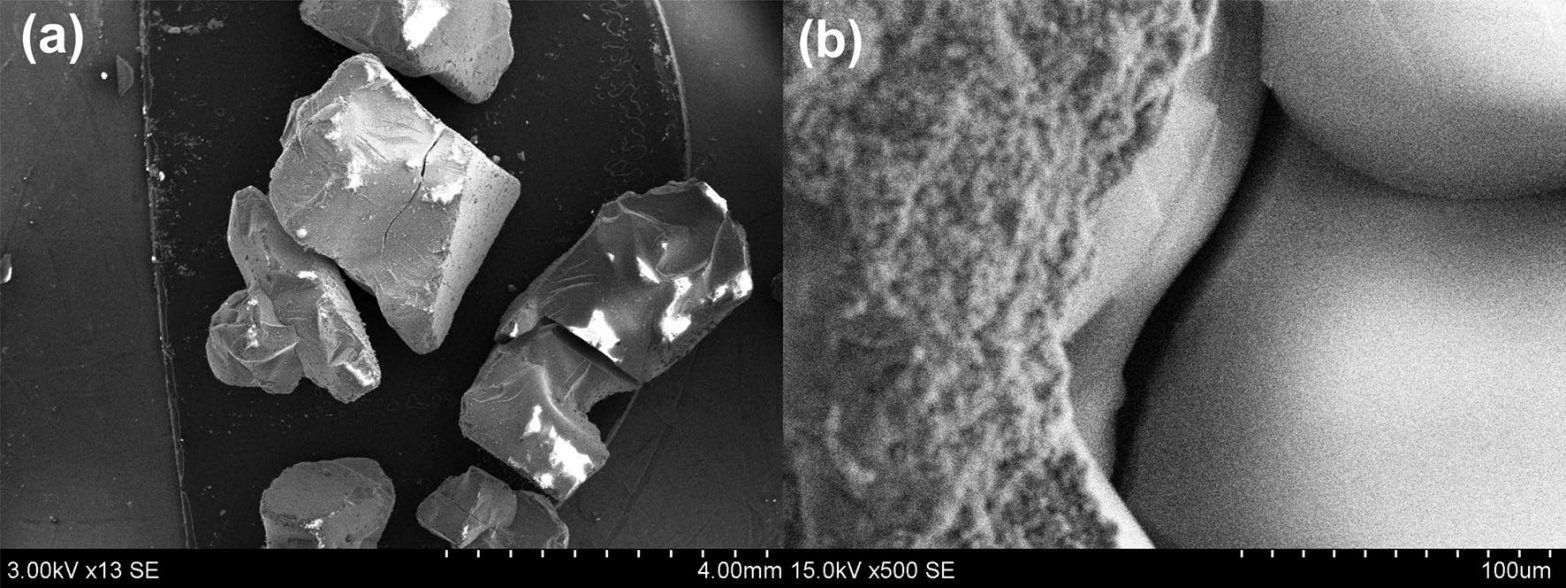
(**a**) Cracks on the surface of native silica aerogel; (**b**) cracks aerogel in the hydrated concrete specimen.

The plain image of hydrophobic silica aerogel in Fig. 5a suggested that it is a very brittle material with cracks on the surface that can easily break into pieces during the mixing process and lead to lower mechanical properties. Moreover, in Fig. 5b, the cracked surface of aerogel particles in the hydrated concrete specimens can be clearly noticed. Literatures^20,49,50^ also indicate the brittleness of aerogel and a decrease in the mechanical performances with the incorporation of aerogel.

**Figure 6:**
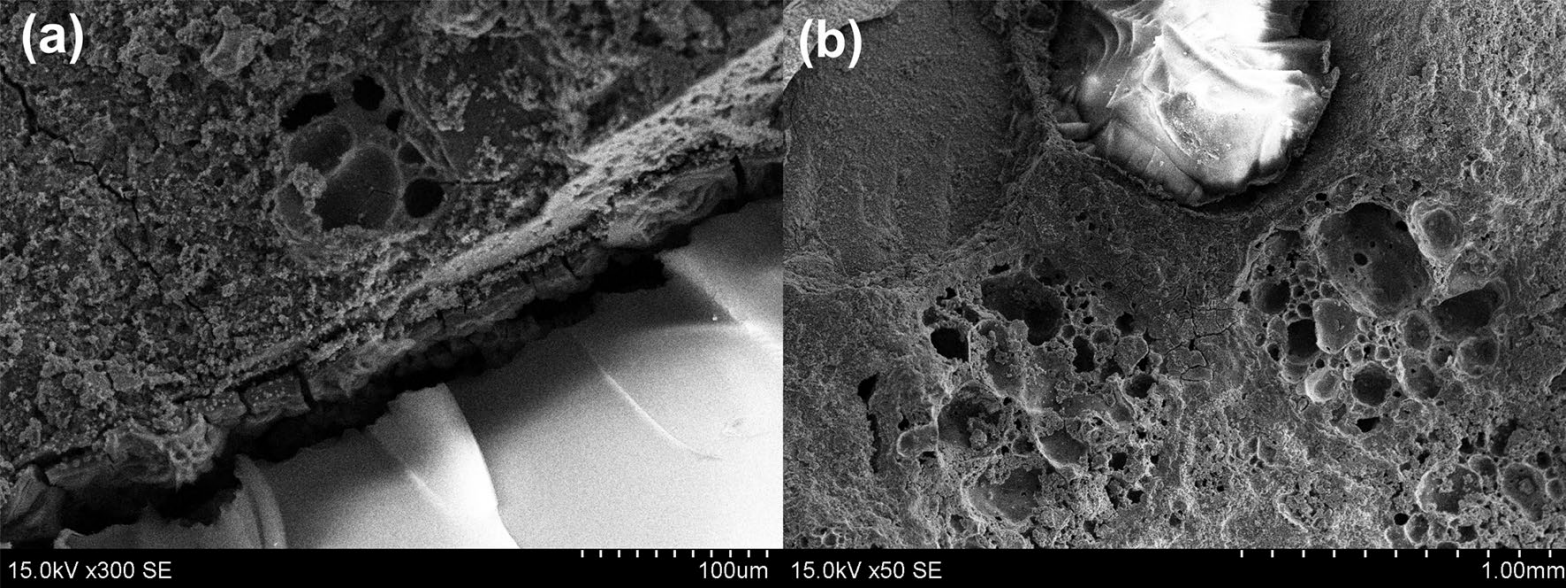
(**a**) Separation gaps between aerogel and cementitious material in the transition zone, (**b**) good adhesion of expanded glass aggregates with cementitious material.

**Figure 7:**
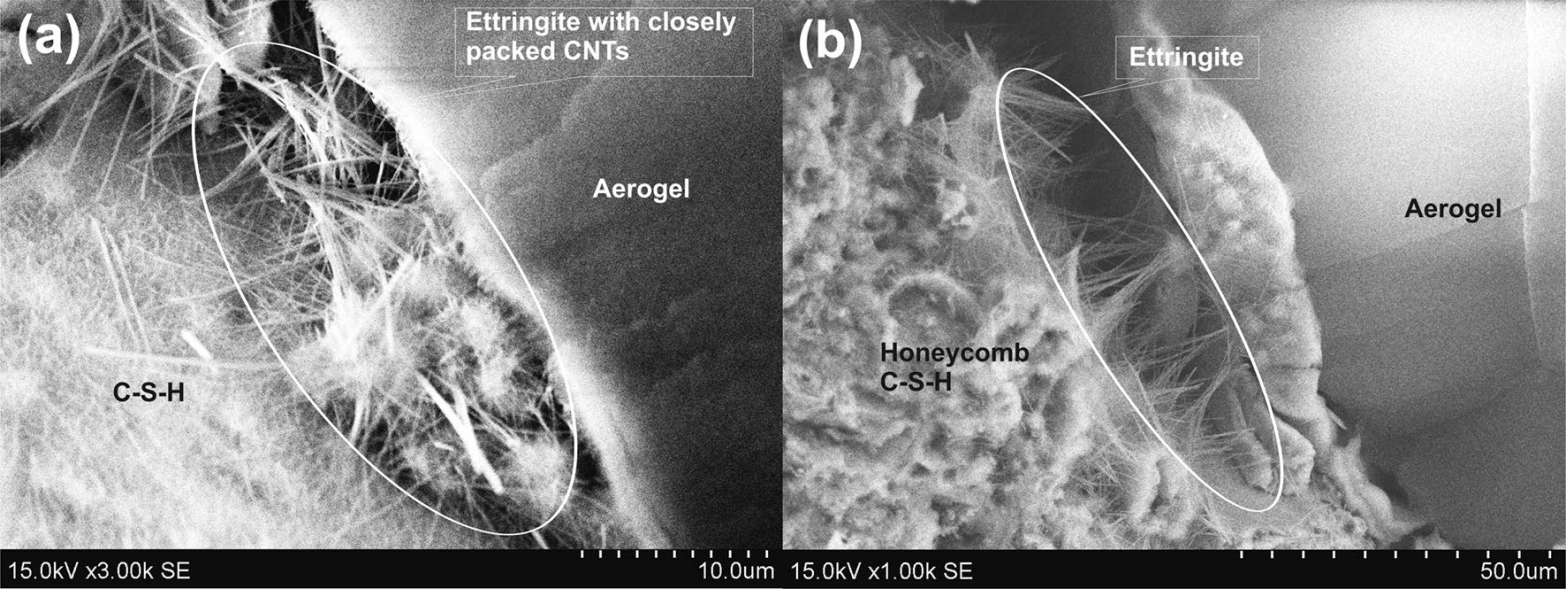
Agglomeration of CNTs and presence of ettringite and honeycomb structure of C–S–H gel in the transition zone of aerogel and cementitious material.

Due to the hydrophobic nature of silica aerogel, it do not develop chemical bonds with the hydrophilic cement matrix. Figure 6a clearly shows the separation gaps between aerogel and surrounding cementitious material in the transition zone of the control sample. This phenomenon indicates the lower adhesion properties of hydrophobic silica aerogel. Similar separation gaps were identified by Gao et al.^31^ and Adhikary et al.^20^. While better adhesion was observed for expanded glass aggregates with cementitious materials, as indicated in Fig. 6b. Through the separation gaps, air and/or water can easily transport and makes concrete weaker. Interestingly the separation gaps were reduced by utilizing carbon nanotubes as indicated in Fig. 7. Separation gaps between hydrophobic silica aerogel and surrounding cement-based materials were filled by hydration products and CNTs.

**Figure 8:**
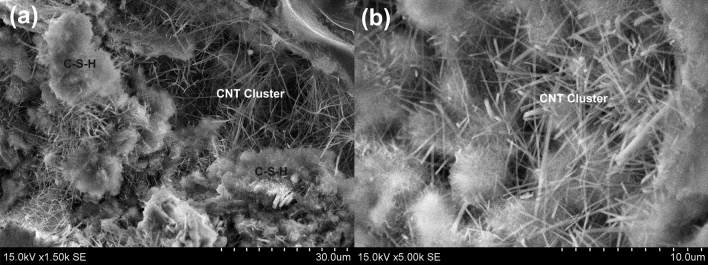
The well-dispersed concrete sample under high concentration of CNT at different magnification; the presence of C–S–H gel surrounding to the CNTs.

**Figure 9:**
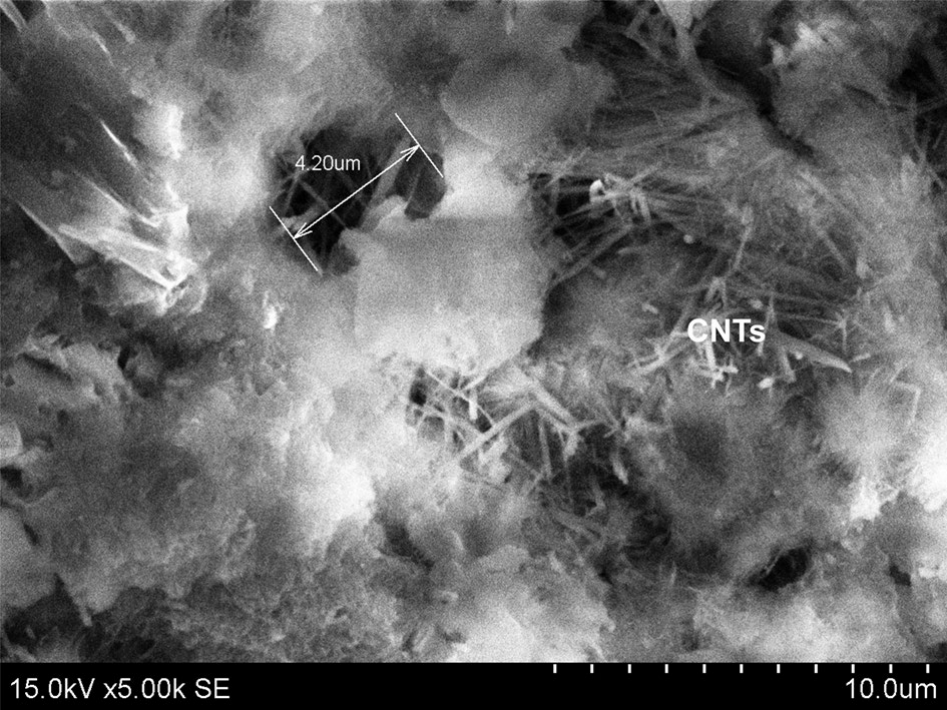
CNTs filling the micropores of the lightweight concrete structure.

SEM image of CNT-LWAC, as shown in Fig. 8 indicates that the CNTs were almost dispersed uniformly within the concrete structure. At the high concentration of CNTs, a network-like distribution within the composite structure was noticed. Zou et al.^8^, Vesmawala et al.^5^, and Collins et al.^10^ also reported that CNTs could be effectively dispersed within the concrete structure by ultrasonication energy and polycarboxylate superplasticizer, may be due to this fact high agglomeration of CNTs was not noticed. Unlikely in the present study, the needle-like structure of ettringite was observed along with the agglomeration of CNTs in the transition zone of aerogel, as shown in Fig. 7. However, increasing the duration of ultrasonication and concentration of superplasticizer can effectively help to improve the dispersion of CNTs without agglomeration. Moreover, CNTs were found to reinforce the concrete structure's micropores, as indicated in Fig. 9.

Zhu et al.^45^ suggested that due to the hydration process of cementitious materials, aerogel particles can slightly react with the pore solution and get partially dissolved in an alkaline environment to form C–S–H with a low Ca/Si ratio. de Fátima Júlio et al.^51^ and Hai-li et al.^52^ reported that aerogel particles could promote hydration due to high surface activity that leads to ASR, and Si–O–Si might form C–S–H. Incorporation of CNTs to the cement composite also provides sites for the formation of calcium silicate hydrate (C–S–H) by acting as a nucleating agent^53,54^. The honeycomb structure of C–S–H gel and its presence near to the CNTs can easily be observed in Figs. 7a and 8b, which illustrates the nucleating effects of CNTs.

### X-ray diffraction analysis of CNT-LWAC

**Figure 10:**
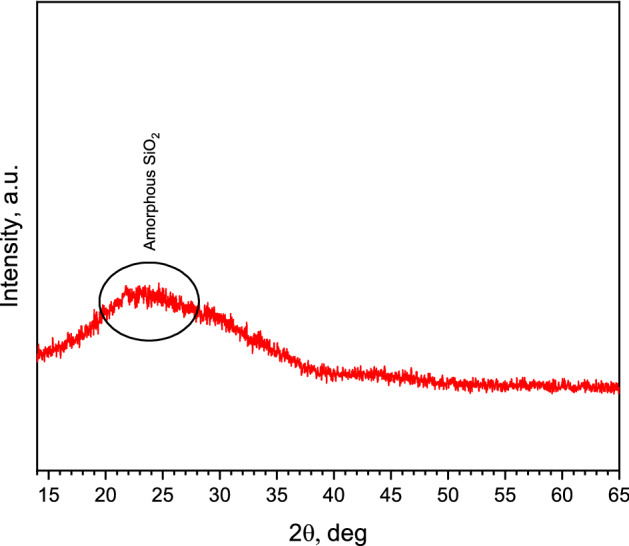
X-ray diffraction pattern of expanded glass aggregates.

**Figure 11:**
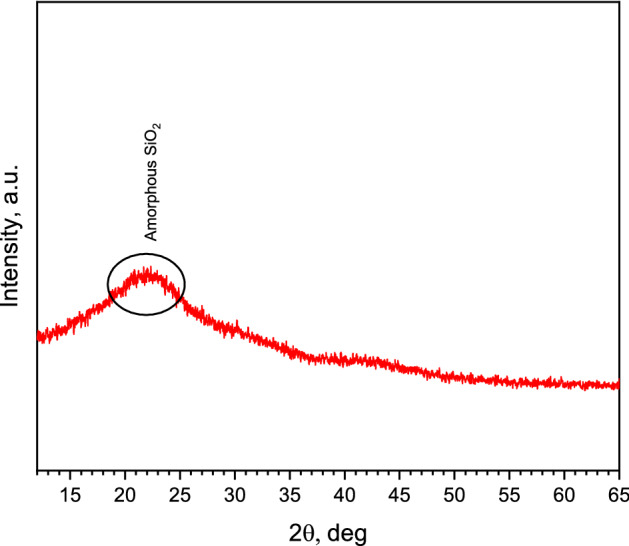
X-ray diffraction pattern of silica aerogel.

**Figure 12:**
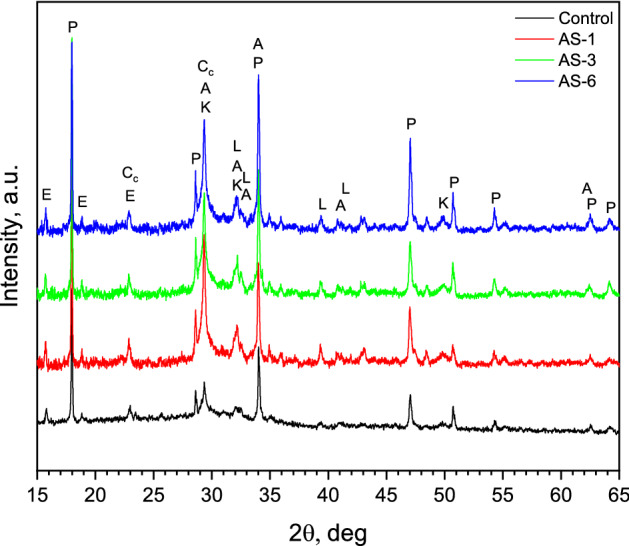
X-ray diffraction pattern of sonicated CNT-LWAC specimens. A—alite Ca_54_MgAl_2_Si_16_O_90_ (13–272); E—ettringite Ca_6_Al_2_(SO_4_)_3_(OH)_12_·26H_2_O (41–1451); L—Ca_2_SiO_4_ belite (33–302); K—Ca_1.5_Si O_3.5_∙× H_2_O calcium silicate hydrate (33–306), Cc—Ca(CO)_3_ calcite (24–27); P—Ca(OH)_2_ portlandite (1–837).

Figures 10, 11, and 12 show the X-ray diffraction analysis of expanded glass aggregates, silica aerogel, and LWAC specimens (control, AS-1, AS-3, and AS-6), respectively. X-ray diffraction pattern of silica aerogel reveals the amorphous nature of silica aerogel and expanded glass aggregates. In Figs. 10 and 11, a hump was observed for the amorphous matrix of SiO_2_ at around 2θ = 22 and 23 °C for silica aerogel and expanded glass aggregates, respectively. In Fig. 12, CNT-LWAC concrete specimen illustrates that the intensity of portlandite near about 34° and 47° increases with the increasing concentration of CNT. The increasing peak of calcium silicate hydrate near 29°, 32°, and 50° explained the nucleation effects of carbon nanotubes. Moreover, CNT-LWAC shows a slightly higher amount of calcite and ettringite than the control specimen. However, a higher amount of hydration products was observed for CNT incorporated LWAC specimens. A higher concentration of CNTs within the concrete structure leads to higher growth of hydration products. A similar increasing peak of hydration products by incorporating CNT in the cementitious composite was identified by El-Gamal et al.^55^.

## Materials and methods

### Materials used in CNT-LWAC

For the preparation of lightweight concrete specimens, ordinary Portland cement (OPC) of grade CEM I 42.4R was used as binding materials according to EN 197-1:2011^56^ standard, and 50 μm size (average particle size) zeolite powder was used as a pozzolanic additive. A total of 500 kg/m^3^ binding materials was used to prepare lightweight concrete samples, where 90% volume consists of cement and 10% consists of zeolite. The chemical composition of cement and zeolite are shown in Table 1. The standard EN 13055-1:2002/AC:2004^57^ was followed for the combinations of four different sizes of expanded glass aggregates, and 1–2 mm size irregular shaped hydrophobic silica aerogel particles with bulk density 70 kg/m^3^ holding the approval No. Z-3.212-1948 from the DIBt—German Institute of Construction Technology were used as lightweight aggregates. The concrete specimens were prepared with 45% volume of total aggregates of silica aerogel having 70 kg/m^3^ bulk density while the rest of 55% of aggregate contains a combination of 1–2 mm, 0.5–1 mm, 0.25–0.50 mm, and 0.1–0.3 mm size expanded glass aggregates. The physical properties of expanded glass aggregates are shown in Table 2. Polycarboxylate ether polymer-based superplasticizer (1.8% of cement mass) and stabilizer (0.3% of cement mass) were used as chemical admixtures. Powder-type MWCNT supplied by Advanced 2D Materials Co. Ltd. was used in the study. The MWCNT was black in color with an inner diameter of 3–5 nm and the outer diameter of 8–15 nm; and lengths of 3–12 μm. The specific surface area was higher than 233 m^2^/g, and density was approximately 0.15 g/cm^3^. Resistivity was observed at 1412 μΩm. MWCNT was synthesized by the chemical vapor deposition (CVD) technique. Multiwall-carbon nanotubes (MWCNTs) were used as nanofibers to enhance the mechanical performance of the lightweight aggregate concrete. The properties of MWCNTs are shown in Table 3.

**Table 1:** Chemical properties of OPC and zeolite.

Chemical composition	CaO	SiO_2_	Al_2_O_3_	Fe_2_O_3_	MgO	K_2_O	Na_2_O	Na_2_O eq	SO_3_	CI	TiO_2_	LOI	Insoluble residue	Free Lime	Lime stone
OPC	63	20.4	4.1	3.5	2.9	0.7	0.23	0.74	3.2	0.03	–	2.5	0.5	1.2	3.9
Zeolite	2.8	58.7	9.0	1.4	0.7	2.6	–	–	0.1		0.2	5.1	–	–	–

**Table 2:** Physical properties of expanded glass aggregates.

Designation	Standard	Expanded glass aggregate size			
		0.1–0.3 mm	0.25–0.50 mm	0.5–1 mm	1–2 mm
Bulk density in kg/m^3^	EN 1097–3	400	340	270	230
Compressive strength (± 15%)	EN 13055-1, A annex	2.8	2.5	2.3	2
Thermal conductivity in W/(m–k) (± 0.02)	EN 12939:2002	0.0767	0.0767	0.0713	0.0663
WATER absorption % by mass (absorption % after 24 h submerged in water)	EN 1097-6:2002, C annex	25	25	20	20
Specific density		2.3	2.3	2.3	2.3
pH value		9–11			
Softening point		~ 700 °C/1300 °F			
Color		Cream white

**Table 3:** Properties of multiwalled carbon nanotubes (MWCNTs).

Material type	Appearance	Purity	Inner diameter	Outer diameter	Length	Specific surface area	Density	Actual density	Resistivity	Preparation method
MWCNT	Black powder	> 95 wt%	3–5 nm	8–15 nm	3–12 μm	> 233 m^2^/g	0.15 g/cm^3^	2.1 g/cm^3^	1412 μΩm	CVD

### Dispersion of CNTs and specimen preparation

The dispersion of carbon nanotubes in the cement matrix is more challenging than in the conventional concrete mixture. Due to the reliable van der Waals forces between carbon nanotubes, it is necessary to maintain the separation of aggregated carbon nanotube bundles to protect cement composites from defects. In this study, carbon nanotubes were sonicated separately in water by ultrasonication energy (in 40% of total water content) with polycarboxylate based superplasticizer for 3 min. Ultrasonic treatment was carried out by Bandelin Electronic ultrasonic converter UW 3400 of 200 W power and 20 kHz frequency. After mixing lightweight concrete composition with 60% total water, sonicated carbon nanotubes liquid was added to the concrete mixture and manually mixed for another 5 min. After the final mixing process, the flowability test was carried out, and concrete samples were molded into 16 × 4 × 4 cm size prisms and kept at room temperature for 24 h for the hardening process. After the setting process, concrete samples were demolded and kept immersed in water until the 28th day of the hydration process in the climatic chamber, having more than 95% RH and 20 ± 1 °C temperature. The mixing composition of the lightweight concrete samples is shown in Table 4.

**Table 4:** Mixing composition of lightweight concrete samples, materials for 1 m^3^ of concrete.

Series	Cement, kg	Aerogel, kg	CNT%	CNT, kg	Aggregate (1/2 + 1/0.5 + 0.5/0.25 + 0.01/0.3), kg	Zeolite, kg	Superplasticizer, kg	Stabilizer, kg	Water w/b = 0.65	Sonication time ( min)
Control	454.5	31.5	0	–	34.5 + 54 + 34 + 40	45.45	8.181	1.3635	325	–
AS-1	454.5	31.5	0.04	0.1818	34.5 + 54 + 34 + 40	45.45	8.181	1.3635	325	3
AS-2	454.5	31.5	0.10	0.4544	34.5 + 54 + 34 + 40	45.45	8.181	1.3635	325	3
AS-3	454.5	31.5	0.15	0.6816	34.5 + 54 + 34 + 40	45.45	8.181	1.3635	325	3
AS-4	454.5	31.5	0.30	1.3632	34.5 + 54 + 34 + 40	45.45	8.181	1.3635	325	3
AS-5	454.5	31.5	0.45	2.0453	34.5 + 54 + 34 + 40	45.45	8.181	1.3635	325	3
AS-6	454.5	31.5	0.60	2.7264	34.5 + 54 + 34 + 40	45.45	8.181	1.3635	325	3

### Methods

The flowability of the CNT-LWAC was performed by flow table test satisfying EN 12350-5:2009^58^ standard requirements. For each type of concrete specimen, three times a flow table test was performed, and the mean value was taken as a result.

Compressive strength of CNT-LWAC was measured on the 28th day of the curing process, satisfying BS EN 196-1:2016^59^ standard requirements. The mean value of three specimens was taken as a result of each type of concrete sample.

Water absorption kinetics observation of the lightweight aggregate concrete specimens reveals the increase in water absorption rate on the 28th day of hydration. LWAC specimens were oven-dried at 105 °C after the 28 days of hydration and immersed in water for 15 min, 1 h, 24 h, and 48 h to observe water absorption in the LWAC specimens.

To perform a semi-adiabatic calorimetry test, several mortar samples were prepared with cement and aerogel containing different concentrations of CNTs. CNTs were sonicated in water for 3 min before mixing with the cement. Table 5 shows the mixing composition of cement paste.

**Table 5:** Mixing composition of CNT-cement paste for the semi-adiabatic calorimetry test.

Sample	Cement (g)	CNT (g)	Water (g)	Aerogel (g)
SA-1	100	–	35	
SA-2	100	–	35	31.5
SA-3	100	0.04	35	31.5
SA-4	100	0.15	35	31.5

Scanning electronic microscopy of CNT-LWAC was analyzed by a high-resolution electronic microscope FEI Quanta 200 FEG with Schottky field emission gun (FEG), an energy-dispersive X-ray spectrometer (EDS) with a silicon type drift droplet detector.

The X-ray diffraction analysis of the composite specimens was performed with X-ray diffractometer DRON-6 with Bragg–Brentano geometry using Ni-filtered Cu Kα radiation and graphite monochromator, operating with the voltage of 30 kV and emission current of 20 mA. The step-scan was performed from the angular range 2°–70° (2θ), and each step of 2θ was 0.02°.

## Conclusion

Study results analyses the viability of using MWCNTs to improve the compressive strength of lightweight aggregate concrete prepared by silica aerogel and expanded glass aggregates. The following conclusions can be drawn based on the obtained results.

- The flowability of CNTLWAC was decreased by increasing the concentration of CNTs. At 0.6 wt% of CNT loading, an almost 17% reduction in flowability was measured for the lightweight concrete specimen.
- The utilization of CNTs significantly improved the compressive strength of the aerogel added lightweight concrete. Almost 41% improvement in the strength of lightweight concrete was observed at 0.6 wt% CNTs loading.
- The dispersion technique of CNTs by sonication with water and polycarboxylate based superplasticizer worked almost effectively to disperse the CNTs within the concrete structure. However, some agglomerations were identified in the transition zone of aerogel.
- CNTs were found to promote the growth of C–S–H, portlandite, and calcite. The study revealed the presence of more hydration products surrounding the CNTs.
- Separation gaps were identified in the transition zone between the hydrophobic silica aerogel and cementitious materials for the control specimen. The utilization of CNTs effectively helped to reduce the separation gaps by filling the voids and gaps. Moreover, the nucleating effects of CNTs were noticed; separations gaps and voids were not only filled by CNTs, but hydration products were also found in the gaps.
